# Associations of Moderate-to-vigorous Physical Activity and Sitting Time With Risk of Disability and Mortality Among Japanese Older Adults

**DOI:** 10.2188/jea.JE20240385

**Published:** 2025-09-05

**Authors:** Daiki Watanabe, Tsukasa Yoshida, Yuya Watanabe, Yosuke Yamada, Motohiko Miyachi, Misaka Kimura

**Affiliations:** 1 Faculty of Sport Sciences, Waseda University, Saitama, Japan; 2 National Institute of Health and Nutrition, National Institutes of Biomedical Innovation, Health and Nutrition, Osaka, Japan; 3 Institute for Active Health, Kyoto University of Advanced Science, Kyoto, Japan; 4 Senior Citizen’s Welfare Section, Kameoka City Government, Kyoto, Japan; 5 National Institute of Biomedical Innovation, National Institutes of Biomedical Innovation, Health and Nutrition, Osaka, Japan; 6 Faculty of Sport Study, Biwako Seikei Sport College, Shiga, Japan; 7 Sports and Health Sciences, Graduate School of Biomedical Engineering, Tohoku University, Miyagi, Japan; 8 Laboratory of Applied Health Sciences, Kyoto Prefectural University of Medicine, Kyoto, Japan

**Keywords:** International Physical Activity Questionnaire-Short Form, long-term care insurance, sedentary behavior, relative excess risk due to interaction, Switch 10

## Abstract

**Background:**

The interaction and substitution effects of physical activity (PA) and sitting time (ST) living in non-western countries have not been well investigated. This study aimed to examine the association of moderate-to-vigorous physical activity (MVPA) and ST with disability and mortality in older adults.

**Methods:**

This prospective study analyzed data from 10,164 adults aged over 65 years who participated in the Kyoto-Kameoka study in Japan. We evaluated MVPA and ST using the validated International Physical Activity Questionnaire-Short Form. Participants were categorized into four groups based on their levels of MVPA (150 min/week) and ST (300 min/day): low MVPA/high ST, low MVPA/low ST, high MVPA/high ST, and high MVPA/low ST. Outcomes were gathered between July 30, 2011, and November 30, 2016.

**Results:**

Over a median follow-up of 5.3 years (45,461 person-years), 2,273 disability cases were documented. The low MVPA/high ST groups were associated with higher disability risk than those in the high MVPA/low ST groups (hazard ratio [HR] 1.52; 95% confidence interval [CI], 1.31–1.75), and the interaction between MVPA and ST accounted for 48.5% of the relative excess risk of disability in the low MVPA/high ST group (*P* for interaction = 0.006). Daily replacement of 10 minutes of ST with 10 minutes of MVPA was associated with a reduced risk of disability (HR 0.980; 95% CI, 0.971–0.989) and all-cause mortality (HR 0.975; 95% CI, 0.962–0.988).

**Conclusion:**

These findings indicate that even a small substitution of ST with MVPA could help lower both the risk of disability and mortality.

## INTRODUCTION

Physical inactivity is a modifiable cause of shortened lifespans.^1^ Its prevalence is one in three or four adults and has increased over time, especially in high-income countries.^2^ Recent physical activity (PA) guidelines to promote good health have emphasized engaging in moderate-to-vigorous physical activity (MVPA) and reducing sedentary behavior.^3^ However, many people experience decreased MVPA, and their sedentary behavior increases with aging.^4^ While prolonging their lifespan is important for older adults, appropriate lifestyle, including MVPA and sedentary behavior status, to ensure the preservation of the capacity to live independently and function well during late life is of even greater significance.^5^

MVPA and sitting time (ST) are independently associated with increased risk of mortality, and it has been shown that those who have high ST and low MVPA have a significantly higher mortality risk.^6^^,^^7^ Therefore, understanding interactions between low MVPA and high ST are more useful in evaluating public health benefits because they provide more insight into which subgroups might be best served by treatment or intervention.^8^ In addition, clarifying the dose-response relationship between MVPA or ST and outcomes for each subgroup provides knowledge of the target values for MVPA or ST for each group.

Since an individual’s daily schedule consists of sleep, sedentary behavior, and physical activity, increasing the time spent on one activity inevitably reduces the time available for others.^9^ To address this, the isotemporal substitution model has been introduced as an effective method to examine the impact of replacing one activity with another for an equivalent duration.^10^ Several epidemiological studies using this model have indicated that the replacement of ST or sedentary behavior with MVPA is inversely associated with all-cause mortality among adults living in Western countries.^11^^–^^17^ However, replacing ST with MVPA could result in different health effects^11^^–^^17^ because MVPA and ST status vary among cultures and countries.^18^ It is especially necessary to evaluate the substitution effects of ST with MVPA in reducing both the risk of death and disability in older Japanese people because Japanese people have been shown to have the longest ST, as assessed using a questionnaire among people in 20 countries.^19^ This information may contribute to extending the healthy lifespan of older adults.

In this study, we aimed to (1) evaluate the association of the interaction between MVPA and ST with disability and (2) examine the replacement of ST with MVPA with the risk of disability and mortality in older adults. Building on our previous findings, which highlighted an association between the interaction of MVPA and ST with mortality in this cohort,^6^ we hypothesized that (1) the combination of low MVPA and high ST was strongly associated with the risk of disability through the interaction of these factors, and that (2) the slight replacement of ST with MVPA may be beneficial in both a reduction in the disability and mortality risk.

## METHODS

### Study population

**Figure 1.**
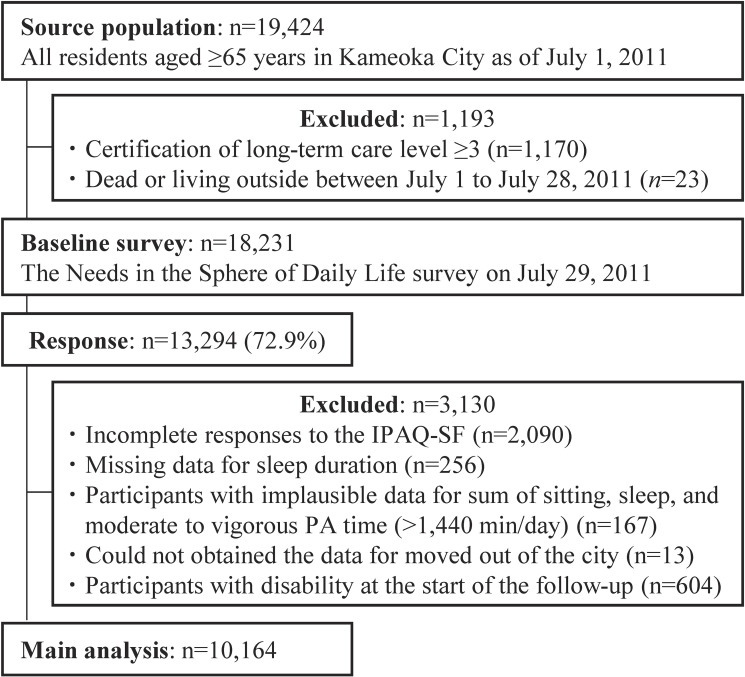
Participant flow diagram. IPAQ-SF, International Physical Activity Questionnaire - Short Form; PA, physical activity.

We utilized data from a population-based Kyoto-Kameoka prospective cohort study, targeting adults aged 65 years and older residing in Kameoka, Kyoto, Japan.^6^^,^^20^^–^^24^ The study was initiated with a mail survey conducted on July 29, 2011, targeting all residents aged 65 years and older living in Kameoka City as of July 1, 2011. Eligible participants were identified by a city official based on the Basic Resident Register, which includes residents’ names, dates of birth, and sex (Figure 1). The baseline survey incorporated the International Physical Activity Questionnaire-Short Form (IPAQ-SF) to assess MVPA and ST. Of 18,231 eligible residents, 13,294 responded to the survey, yielding a response rate of 72.9%.

Among the 13,294 respondents, we excluded participants with incomplete IPAQ-SF responses (*n* = 2,090); those with missing data for sleep duration (*n* = 256); those with implausible data for the sum of sitting, sleep, and MVPA time (>1,440 min/day) (*n* = 167); those who had moved out of Kameoka City (*n* = 13); and those with a disability at the beginning of the follow-up period (*n* = 604). A total of 10,164 participants (5,366 women and 4,798 men) were included in the analysis.

The Research Ethics Committee approved the study. Informed consent was obtained from all participants through a mail survey.

### Evaluation of MVPA and ST

Self-reported MVPA and ST, excluding sleep over the previous week, were assessed using the IPAQ-SF. This tool has been validated for reliability across 12 countries, including Japan.^18^ Specifically, in older Japanese adults, MVPA estimated via IPAQ-SF demonstrated Spearman correlation coefficients of 0.43–0.54 when compared with accelerometer-derived MVPA, while ST showed intraclass correlation coefficients of 0.66–0.82 for reproducibility based on measurements taken two weeks apart.^25^ The IPAQ-SF focuses on MVPA conducted in bouts lasting at least 10 minutes per session. PA duration was calculated for activities achieving ≥3.0 metabolic equivalents (METs).

### Outcomes

The incidence of disability was assessed using the long-term care insurance systems in Japan, which classifies disability (≥support level 1) as requiring support with instrumental activities of daily living.^22^^,^^26^ Mortality data was obtained using the Basic Resident Register records. Both disability and survival data were provided by city officials and obtained between July 30, 2011, and November 30, 2016. Participants whose administrative records were removed or relocated outside the municipality during the study period were censored.

### Statistical analysis

Participants were categorized into four groups based on their levels of MVPA and ST according to the previous study as following^6^: low MVPA/high ST (MVPA: <150 min/week and ST: ≥300 min/day; *n* = 3,505), low MVPA/low ST (MVPA: <150 min/week and ST: <300 min/day; *n* = 3,211), High MVPA/high ST (MVPA: ≥150 min/week and ST: ≥300 min/day; *n* = 1,478), and high MVPA/low ST (MVPA: ≥150 min/week and ST: <300 min/day; *n* = 1,970). These cutoff values for PA and ST have been used in our previous study,^6^ the WHO PA guideline 2020,^3^ and previous studies.^27^ Missing indicators were created if covariates pertaining to body mass index (BMI) (*n* = 524; 5.2%), smoking status (*n* = 394; 3.9%), alcohol drinking status (*n* = 333; 3.3%), family structure (*n* = 732; 7.2%), education attainment (*n* = 1,105; 10.9%), socioeconomic status (*n* = 430; 4.2%), denture use (*n* = 236; 2.3%), medications (*n* = 721; 7.1%), and frailty status (*n* = 1,092; 10.7%) were missing. Details of covariate assessment are provided in eMaterial 1.

During follow-up periods, each individual’s person-years were calculated from the date their MVPA and ST status was assessed to the earliest occurrence of disability, death, relocation out of the study area, or the end of the follow-up period. Disability and mortality rates for the MVPA/ST status category were expressed as the events per 1,000 person-years. To adjust for confounding factors related to MVPA/ST status, disability, and mortality, we performed a multivariate Cox proportional hazards model, incorporating covariates. The Schoenfeld residual test verified the proportional hazards assumption (*P* = 0.153). The results are reported as hazard ratios (HRs) with 95% confidence intervals (CIs), using the high MVPA/low ST group as the reference category. To assess the interaction between ST, MVPA status, and disability, we calculated additive interactions using the relative excess risk due to interaction (RERI). We applied a restricted cubic spline model with three knots based on the baseline MVPA and ST status distribution to evaluate the target value of MVPA and ST curvilinear relationship between MVPA, ST, and disability. To address data sparsity, the analysis was truncated at 309 minutes/day of MVPA, which corresponds to the 99th percentile of the distribution. The results are expressed as HRs with 95% CIs, using a baseline MVPA and ST duration of 0 minutes/day as the reference.^6^ The linear trend *P*-value was calculated by treating MVPA and ST as continuous variables. To assess the statistical significance of nonlinearity, Wald’s test was used to compare the likelihood ratio of the spline model against a linear model. A *P*-value <0.05 was considered indicative of a significant non-linear relationship between the exposure (MVPA or ST) and the outcome (disability).^20^

The risk of disability and mortality associated with replacing daily 10 minutes of ST duration with 10 minutes of MVPA duration was calculated using the multivariate-adjusted substitution method. This analysis was conducted using a leave-one-out model,^10^ where the models included the substitute (MVPA duration), fix (sleep duration and other [other duration = 1,440 (min) − sleep (min) − ST (min) − MVPA (min)]), and substitution time (ST duration). The resulting HRs and 95% CI of a 10-minute/day substitution were interpreted as the estimated risks of disability and mortality associated with increasing MVPA duration while simultaneously reducing the equivalent amount of ST.

The following three types of sensitivity analyses were performed: 1) we excluded disability events (423 women and 216 men) recorded in the first year of follow-up; 2) we conducted a similar analysis using a dataset in which missing covariate values were supplemented using multiple imputations; and 3) to address the limitations of the cause-specific hazard model, which treats competing events as censored, we performed a multivariable sub-distribution hazard model.

A multivariate analysis was performed using the two models according to confounding factors reported in previous studies.^11^^–^^17^ Model 1 included adjustments for age, sex, and population density. Model 2 included adjustments for all variables in model 1, along with BMI, alcohol consumption, smoking status, self-reported economic status, family structure, education level, sleep duration, medication use, denture use, number of comorbidities, and frailty. When using the missing indicator method, it is necessary to create indicators for missing values, so continuous variables, such as BMI, sleep duration, and medication use, were converted to categorical variables. The classification method for categorical variables followed the previous study,^24^ and detailed classification is described in eMaterial 1.

For all analyses, a two-tailed probability of less than 5% was considered statistically significant. Statistical analyses were conducted using STATA MP, Version 15.0 (StataCorp LP, College Station, TX, USA).

## RESULTS

Table 1 displays the baseline characteristics of the people grouped by MVPA and ST status. The mean MVPA and ST for the entire population were 32 and 317 minutes/day, respectively. The high PA/low ST group was younger and had a lower percentage of women, more alcohol drinkers, higher educational attainment, and lower frailty than the low MVPA/high ST group.

**Table 1.** Baseline study participants characteristics by physical activity and sitting time status

	Total (*n* = 10,164)		Moderate to vigorous physical activity and sitting time status							

			HPA/LST (*n* = 1,970)		HPA/HST (*n* = 1,478)		LPA/LST (*n* = 3,211)		LPA/HST (*n* = 3,505)	
Age, years^a^	73.6	(6.5)	71.4	(5.2)	72.1	(5.6)	73.4	(6.1)	75.6	(7.1)
Women, *n* (%)^b^	5,366	(52.8)	842	(42.7)	682	(46.1)	1,808	(56.3)	2,034	(58.0)
PD ≥1,000 people/km^2^, *n* (%)^b^	4,552	(44.8)	940	(47.7)	692	(46.8)	1,369	(42.6)	1,551	(44.3)
Body mass index, kg/m^2 a^	22.6	(3.4)	22.7	(3.2)	22.6	(3.2)	22.5	(3.4)	22.6	(3.7)
Current smoker, *n* (%)^b^	1,132	(11.1)	210	(10.7)	176	(11.9)	373	(11.6)	373	(10.6)
Alcohol drinker, *n* (%)^b^	6,279	(61.8)	1,378	(69.9)	1,015	(68.7)	1,923	(59.9)	1,963	(56.0)
Living alone, *n* (%)^b^	1,142	(11.2)	167	(8.5)	198	(13.4)	322	(10.3)	455	(13.0)
Education ≥13 years, *n* (%)^b^	2,042	(20.1)	498	(25.3)	341	(23.1)	600	(18.7)	603	(17.2)
HSES, *n* (%)^b^	3,297	(32.4)	673	(34.2)	492	(33.3)	1,037	(32.3)	1,095	(31.2)
Denture use, *n* (%)^b^	6,004	(59.1)	1,103	(56.0)	822	(55.6)	1,953	(60.8)	2,126	(60.7)
No medication, *n* (%)^b^	2,146	(21.1)	520	(26.4)	354	(24.0)	683	(21.3)	589	(16.8)
Hypertension, *n* (%)^b^	3,781	(37.2)	704	(35.7)	527	(35.7)	1,188	(37.0)	1,362	(38.9)
Stroke, *n* (%)^b^	410	(4.0)	64	(3.3)	57	(3.9)	103	(3.2)	186	(5.3)
Heart disease, *n* (%)^b^	1,242	(12.2)	190	(9.6)	161	(10.9)	382	(11.9)	509	(14.5)
Diabetes, *n* (%)^b^	1,029	(10.1)	194	(9.9)	158	(10.7)	303	(9.4)	374	(10.7)
Hyperlipidemia, *n* (%)^b^	939	(9.2)	205	(10.4)	149	(10.1)	271	(8.4)	314	(9.0)
Digestive disease, *n* (%)^b^	479	(4.7)	63	(3.2)	51	(3.5)	141	(4.4)	224	(6.4)
Respiratory disease, *n* (%)^b^	825	(8.1)	116	(5.9)	138	(9.3)	250	(7.8)	321	(9.2)
Urological diseases, *n* (%)^b^	608	(6.0)	120	(6.1)	93	(6.3)	156	(4.9)	239	(6.8)
Cancer, *n* (%)^b^	370	(3.6)	62	(3.2)	60	(4.1)	91	(2.8)	157	(4.5)
Number of chronic diseases^a,c^	0.95	(0.98)	0.87	(0.95)	0.94	(0.96)	0.90	(0.94)	1.05	(1.04)
Frailty, *n* (%)^b^	3,582	(35.2)	347	(17.6)	340	(23.0)	1,105	(34.4)	1,790	(51.1)
Sleep times, min/day^a^	406	(83)	398	(74)	403	(71)	400	(84)	418	(90)
Physical activity, min/week^a^	32	(62)	97	(86)	78	(67)	3	(6)	3	(6)
Sitting times, min/day^a^	317	(215)	162	(62)	440	(158)	159	(63)	496	(207)

HSES, self-reported high socioeconomic status; HST, high sitting time; HPA, high moderate to vigorous physical activity; LPA, low moderate to vigorous physical activity; LST, low sitting time; PD, population density.

Number of missing covariates are as follows: body mass index (*n* = 524; 5.2%), smoking status (*n* = 394; 3.9%), alcohol drinker status (*n* = 333; 3.3%), family structure (*n* = 732; 7.2%), education level (*n* = 1,105; 10.9%), self-reported socioeconomic status (*n* = 430; 4.2%), denture (*n* = 236; 2.3%), medications use (*n* = 721; 7.1%), and frailty (*n* = 1,092; 10.7%).

^a^Values are expressed as mean with standard deviation.

^b^Values are expressed as number with percentage.

^c^The comorbidity scores were calculated by the presence of hypertension, hyperlipidemia, diabetes, stroke, respiratory disease, heart disease, cancer, digestive disease, and urological diseases.

Table 2 indicates the relationship between the MVPA/ST status and disability risk. The median follow-up period was 5.3 years, with a total follow-up time of 45,461 person-years. During this period, 2,273 incident cases of disability (22.4%) were recorded. After adjusting for confounding factors, people in the low MVPA/high ST group had the highest HR for disability compared to those in the high MVPA/low ST group (HR 1.52; 95% CI, 1.31–1.75). These associations weakened slightly but remained statistically significant after excluding individuals who experienced an event in the first year of follow-up (eTable 1). The interaction between MVPA and ST accounted for 48.5% of the excess risk of disability in the low MVPA/high ST group (RERI 0.25; 95% CI, 0.06–0.45; *P* = 0.006). Sensitivity analyses using multiple imputation or sub-distribution hazard models were similar to results as an interaction between MVPA and ST was associated with the disability risk from the main analysis (eTable 2 and eTable 3).

**Table 2.** Association of the interaction between physical activity and sitting time with long-term disability in older Japanese adults

	*n*	Event	PY	Event/1,000 PY		Model 1^a^		Model 2^b^	

				Rate	95% CI	HR	95% CI	HR	95% CI
**MVPA × ST**									
HPA/LST	1,970	239	9,608	24.9	(21.9–28.2)	1.00	(Ref)	1.00	(Ref)
HPA/HST	1,478	223	7,128	31.3	(27.4–35.7)	1.08	(0.90–1.29)	1.04	(0.87–1.25)
LPA/LST	3,211	656	14,666	44.7	(41.4–48.3)	1.31	(1.13–1.52)	1.22	(1.05–1.42)
LPA/HST	3,505	1,155	14,059	82.2	(77.6–87.0)	1.73	(1.50–2.00)	1.52	(1.31–1.75)
*Interaction*^c^									
RERI						0.35	(0.15–0.54)	0.25	(0.06–0.45)
%RERI						47.2%		48.5%	
*P*-value						<0.001		0.006	
**MVPA**									
High	3,448	462	16,737	27.6	(25.2–30.2)	1.00	(Ref)	1.00	(Ref)
Low	6,716	1,811	28,724	63.0	(60.2–66.0)	1.48	(1.33–1.64)	1.35	(1.22–1.50)
**ST**									
Low	5,181	895	24,274	36.9	(34.5–39.4)	1.00	(Ref)	1.00	(Ref)
High	4,983	1,378	21,187	65.0	(61.7–68.6)	1.29	(1.18–1.40)	1.21	(1.11–1.32)

CI, confidence interval; HR, hazard ratio; HST, high sitting time; HPA, high moderate to vigorous physical activity; LPA, low moderate to vigorous physical activity; LST, low sitting time; MVPA; moderate to vigorous physical activity; PY, person-years; Ref, reference; RERI, Relative Excess Risk due to Interaction; ST, sitting times.

^a^Multivariate adjusted model 1 included age, sex, and population density.

^b^Multivariate adjusted model 2 included model 1 and body mass index, alcohol consumption status, smoking status, self-reported economic status, family structure, sleep duration, educational level, denture, medication, number of comorbidities, and frailty.

^c^The additive interaction was calculated as the following equation: RERI = (HR [LPA/HST] − 1) − (HR [LPA/LST] + HR [HPA/HST] − 2). The results are shown as RERI (95% CI).

**Figure 2.**
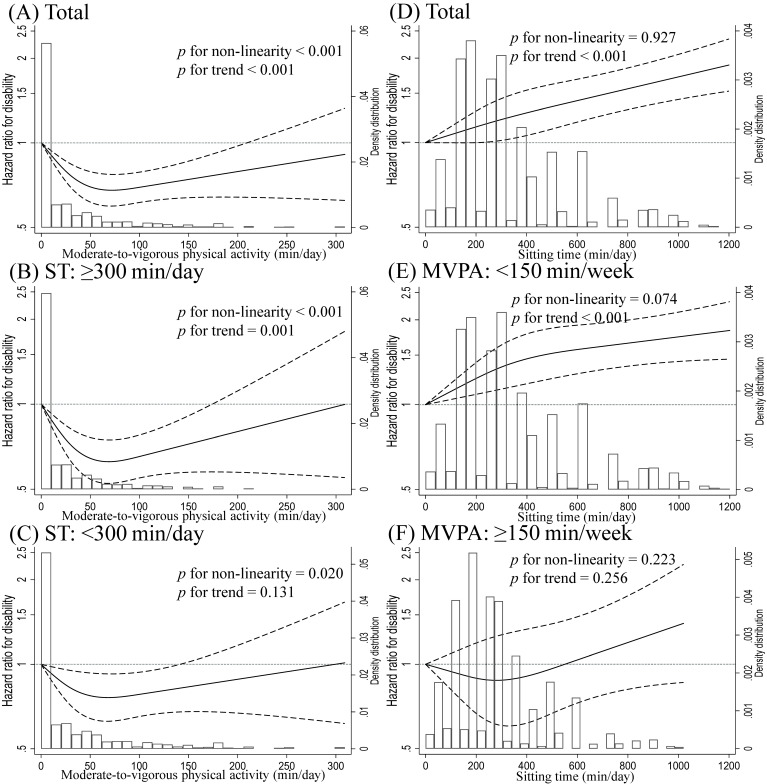
Multivariate adjusted spline model between moderate to vigorous physical activity and sitting time and risk of disability among older adults. Solid lines represent the hazard ratios, while dashed lines indicate 95% confidence intervals (CI). Hazard ratios for disability risk were calculated using the 0 minutes/week of moderate to vigorous physical activity (MVPA) in [**A**] the total participants (*n* = 10,076), [**B**] participants with ≥300 minutes/day of sitting time (ST) (*n* = 4,960), and [**C**] participants with <300 minutes/day of ST (*n* = 5,116) as the reference value and using the 0 minutes/day of ST in [**D**] the total participants (*n* = 10,164), [**E**] participants with <150 minutes/week of MVPA (*n* = 6,716), and [**F**] participants with ≥150 minutes/week of MVPA (*n* = 3,448). The adjustment factors were age, sex, population density, body mass index, alcohol consumption status, smoking status, self-reported economic status, family structure, education level, sleep duration, medication use, denture use, number of comorbidities, and frailty status.

Figure 2 presents the dose-response relationship between MVPA, ST, and disability. In all participants, the range of MVPA associated with the lowest HR for disability was 60–80 minutes per day (*P* for nonlinearity <0.001; Figure 2A). Similar results were observed in the ST status-stratified analyses (Figure 2B and Figure 2C). A log-linear relationship was observed between ST and the disability risk among all participants (*P* for trend <0.001; Figure 2D). In the MVPA status-stratified model, ST was associated with disability in the low MVPA people but not in the high MVPA people (Figure 2E and Figure 2F).

Table 3 shows the association between disability and mortality when replacing the ST with MVPA. Replacing daily 10 minutes of ST with 10 minutes of MVPA was inversely associated with disability (HR 0.980; 95% CI, 0.971–0.989) and mortality (HR 0.975; 95% CI, 0.962–0.988). These relationships were more pronounced in the high ST and low MVPA people than those in the low ST and high MVPA people.

**Table 3.** Hazard ratios of disability and all-cause mortality for replacement sitting times with moderate to vigorous physical activity among older Japanese adults

	Substitution of 10 minutes of sitting time with 10 minutes of MVPA					

	Disability			All-cause mortality		
Person years	45,461			51,061		
Number of events	2,273			1,056		
Rate/1,000 PY (95% CI)	50.0 (48.0–52.1)			20.7 (19.5–22.0)		
	HR	(95% CI)	*P* for trend	HR	(95% CI)	*P* for trend
**Total (*n* = 10,164)**						
Model 1^a^	0.974	(0.965–0.983)	<0.001	0.970	(0.956–0.983)	<0.001
Model 2^b^	0.980	(0.971–0.989)	<0.001	0.975	(0.962–0.988)	<0.001
**ST <300 min/d (*n* = 5,181)**						
Model 1^a^	0.983	(0.967–0.998)	0.028	0.975	(0.953–0.996)	0.022
Model 2^b^	0.986	(0.971–1.001)	0.071	0.977	(0.955–0.999)	0.039
**ST ≥300 min/d (*n* = 4,983)**						
Model 1^a^	0.964	(0.948–0.979)	<0.001	0.940	(0.914–0.966)	<0.001
Model 2^b^	0.973	(0.958–0.988)	<0.001	0.952	(0.927–0.977)	<0.001
**MVPA <150 min/w (*n* = 6,716)**						
Model 1^a^	0.862	(0.771–0.955)	0.004	0.810	(0.669–0.953)	0.009
Model 2^b^	0.902	(0.809–0.997)	0.043	0.835	(0.691–0.980)	0.026
**MVPA ≥150 min/w (*n* = 3,448)**						
Model 1^a^	0.997	(0.986–1.008)	0.586	1.000	(0.983–1.016)	0.954
Model 2^b^	0.997	(0.985–1.008)	0.593	0.995	(0.978–1.012)	0.555

CI, confidence interval; HR, hazard ratio; MVPA; moderate to vigorous physical activity; PY, person-years; ST, sitting times.

^a^Multivariate adjusted model 1 included age, sex, and population density.

^b^Multivariate adjusted model 2 included model 1 and body mass index, alcohol consumption status, smoking status, self-reported economic status, family structure, sleep duration, educational level, denture, medication, number of comorbidities, and frailty.

## DISCUSSION

This study indicated that the interaction between low MVPA and high ST accounted for the RERI of disability in the low MVPA/high ST group. Our findings suggest that replacing 10 minutes of ST daily with 10 minutes of MVPA is associated with a 2% and 2.5% reduction in the HR of disability and all-cause mortality, respectively. To our knowledge, the current study is the first to examine both the interaction and substitution effects of MVPA and ST on the risks of mortality and disability in older adults.

Our results indicate a significant association between low MVPA and high ST and a higher risk of disability. To our knowledge, although these relationships have not yet been reported, several studies have reported ST as a factor that interacts with MVPA to influence health outcomes.^6^^,^^7^ Previous epidemiological studies have indicated that people with high ST might benefit more from increasing MVPA in terms of lower mortality risk than those with low ST,^13^ and our results align with those of these studies. In addition, our spline analysis showed that the MVPA with the lowest HR for disability was 60–80 minutes per day, regardless of ST status. This may provide insight into the creation of target value of MVPA for disability prevention in future PA guidelines. On the other hand, we observed dose-response relationships between ST and disability in the low MVPA group, but no significant association was observed in the high MVPA group. Thus, these results indicate that the adverse effects of prolonged ST on the disability risk depend on MVPA status, and high MVPA partially offsets the sitting time-related disability risk.

In addition, we showed that replacing 10 minutes of ST daily with 10 minutes of MVPA was associated with a 2% and 2.5% reduction in the HR of disability and all-cause mortality, respectively. Previous epidemiological studies have evaluated the effects of replacing ST or sedentary behavior with various types of MVPA on health outcomes. However, the amount of duration replaced has varied across studies. Because the substitution analysis was a linear model, we recalculated the effect sizes from previous studies by replacing 10 minutes of ST daily with 10 minutes of MVPA. To these results, substitution of these durations is associated with decreased HR of death in the following populations: 0.7% and 7.0% in Americans aged 59–82 years with and without ≥2 hours/day of activity, respectively,^11^ 16.3%^12^ and 2.7%^16^ in American adults aged ≥50 years, 1.8% in American adults^14^ and 1.7%^14^ and 3.0%^17^ in British adults aged 37–73 years, 3.7% and 0.5% (walking) in Australian adults with and without ≥6 hours/day of ST, respectively,^13^ and 2.0% in Australian adults aged ≥45 years.^15^ These previous studies, conducted in Western countries, are similar to our findings for older Japanese adults, but there is considerable variation in the effect size in each study (0.5–16.3%).^11^^–^^17^ The variation in effect sizes between these studies may be related to differences in the distribution of PA and ST in the populations. Our study and previous studies have shown that the benefit of replacing ST with MVPA on death risk is greater in those who spend more ST^13^^,^^14^ and less doing MVPA.^11^^,^^16^ Therefore, extrapolation and generalization of these results may require consideration of the distribution of PA and ST in the target populations. In modern societies where individuals are often required to sit for extended times due to work and lifestyle demands, our findings provide additional evidence supporting the benefits of replacing ST with MVPA and could serve as a basis for future public health guidelines and recommendations.^28^

Our results revealed the health benefits of interactions and substitution effects of MVPA and ST. There are two reasons for our findings. First, standing up from a seated position moves the body’s center of gravity to maintain posture and mobilize the leg muscles, including the gastrocnemius, resulting in greater leg muscle contraction than a sitting position.^29^ These increases in heart rate and energy expenditure^30^ explain their inverse associations with mortality risk.^31^ Second, sitting behaviors lead to increased inflammatory markers and blood viscosity levels due to accelerated local (no change in arm vein) coagulation of red blood cells in the leg veins.^32^ This may be associated with reduced vascular endothelial function and increased blood pressure,^33^ resulting from elevated muscle sympathetic nerve activity. These adverse effects have been reduced by interrupting the sitting behavior with low-intensity walking.^34^ These studies support our findings that slightly replacing ST with MVPA reduces disability and mortality risk.

In Japan, the “+10” initiative encourages individuals to increase their daily MVPA by just 10 minutes. A meta-analysis has shown that adding 10 minutes of MVPA per day is associated with a 3.2% relative reduction in the risk of the outcome, including mortality and incidents of lifestyle-related diseases.^35^ Data from the 2010 Japan National Health and Nutrition Survey revealed that 66.8% of individuals aged 60–69 years and 50.8% of those aged 70 years and older reported being able to increase their MVPA by 10 minutes a day.^35^ Based on these findings, our findings suggest that a “Switch 10” approach, which aims to slightly increase MVPA and reduce ST by replacing daily 10 minutes of ST with 10 minutes of MVPA, could be an effective strategy to improve health outcomes, including lifespan and disability risk, especially for older people, even those with low MVPA and high ST.

The strength of the current prospective cohort study is that it evaluated the association between MVPA, ST, disability, and mortality using a validated questionnaire in a large cohort of older adults, consisting of all residents aged 65 years and older living in Kameoka City. However, this study has some limitations. First, the self-reported assessments of MVPA in this study may have included systematic reporting biases. Specifically, MVPA might have been underestimated, as the IPAQ-SF may only capture some types of PA and ST in engaged older adults. Moreover, the association between ST and mortality risk depends on how ST is spent.^36^ Self-reported data may overestimate habitual MVPA levels. However, our results are consistent with those from studies that objectively measured MVPA and ST.^37^ Second, the IPAQ-SF only considers MVPA performed in bouts lasting approximately 10 minutes or more. These results may have underestimated MVPA because MVPA of less than 10 minutes was not assessed. MVPA performed for bouts of <10 minutes has been indicated to be inversely associated with mortality.^38^ Considering the two limitations of the study mentioned above, our results must be re-evaluated using different questionnaires (not bout time) or objective measures of MVPAs and STs. Third, the follow-up period is relatively short. Previously, shorter follow-up studies have suggested a stronger association between PA and mortality, as HRs may change over time.^39^^,^^40^ The results from studies with shorter follow-up periods may be overestimated due to confounding factors related to poor health status and reverse causality.^40^ However, similar results were observed after excluding disability events occurring within the first year of follow-up. Fourth, MVPA and ST assessments were performed only at baseline, meaning individuals’ MVPA/ST status could have changed during the observational period. However, a previous study of Japanese adults aged 26–85 years indicated that the longitudinal trajectory of individual MVPA and inactive time between baseline and approximately 7 years later were well maintained even with aging in both females and males.^4^ The participants in this study may have had stable individual MVPA and ST status rankings during the follow-up period. Finally, while this study adjusted for many confounding factors, residual confounders may still influence the observed relationships between MVPA/ST, mortality, and disability.

In conclusion, this study demonstrates that the interaction between low MVPA and high ST accounts for a relatively high risk of disability in the low MVPA/high ST group. Our findings suggest that slightly replacing ST with MVPA may be beneficial in reducing both the disability and mortality risk, and this benefit was more pronounced in individuals with low MVPA and high ST. These benefits will encourage older adults, including those with low PA or high ST. The effort “Switch 10” of replacing daily 10 minutes of ST with 10 minutes of MVPA may encourage improving health status, including life span in Japan. These findings may be valuable for shaping future PA guidelines.

## SUPPLEMENTARY MATERIAL

eMaterial 1. Other covariates

eTable 1. Results of sensitivity analysis for the relationship between moderate-to-vigorous physical activity and sitting time status and risk of long-term disability after excluding participants with an event in the first year of follow-up

eTable 2. Results of sensitivity analysis for the relationship between moderate-to-vigorous physical activity and sitting time status and risk of long-term disability using a multiple imputation method for missing value of covariates

eTable 3. Results of sensitivity analysis for the relationship between moderate-to-vigorous physical activity and sitting time status and risk of long-term disability using the multivariable sub-distribution hazard model among older adults
