# Supplementary material for: Associations of Moderate-to-vigorous Physical Activity and Sitting Time With Risk of Disability and Mortality Among Japanese Older Adults
Source: J Epidemiol. 2025 Sep 5;35(9):385–92. doi: 10.2188/jea.JE20240385 (PMC12358254; doi:10.2188/jea.JE20240385)
Supplement: Supplementary file 1 [file je-35-385-s001.pdf]

eMaterial 1. Other covariates

All covariates were obtained from the questionnaire data from the baseline survey. We collected data on the following basic characteristics: smoking status ("Do you smoke?": almost daily; sometimes; used to, but quit; never); drinking status ("Do you drink alcohol?": almost daily, sometimes, almost never, never); sleep duration (minutes); living status ("What is your family structure?": living alone, living with family, other); education attainment (years); socioeconomic status ("Economically, how does your life feel currently?": hard, somewhat hard, somewhat easy, easy); oral status ("Do you use dentures?": yes, no); taking medication (number); and chronic disease ("Do you have a disease [presence of hypertension, stroke, heart disease, diabetes, hyperlipidemia, digestive disease, respiratory disease, urological diseases, and cancer]?": yes, no). The comorbidity scores were calculated from the data obtained for the nine comorbidity statuses. The summed value indicated a total score ranging from 0 (no comorbidities) to 9 (poor status). Body mass index was calculated as self-reported body weight divided by height squared (kg/m^2). Frailty was assessed using the validated, self-administered Kihon Checklist consisting of 25 questions. All covariates were used as follows: age (continuous), sex (women or men), population density (≥1,000 or <1,000 people/km^2), body mass index (<18.5, 18.5–21.4, 21.5–24.9, ≥25.0 kg/m^2, missing), smoking status (never smoked, past smoker, current smoker, missing), alcohol consumption (never drank, past drinker, current drinker, missing), family structure (living alone, living with others, missing), education level (≤9, 10–12, ≥13 years, missing), economic status (high, low, missing), sleep duration (<360, 360–<420, 420–<480, ≥480 minutes/day, missing), denture use (yes, no, or missing), medication use (none, 1, 2, 3, 4, ≥5, missing), number of chronic diseases (continuous), and frailty (yes, no, missing).

eTable 1. Results of sensitivity analysis for the relationship between moderate-to-vigorous physical activity and sitting time status and risk of long-term disability after excluding participants with an event in the first year of follow-up

	n	Event	PY	Event/1,000 PY		Model 1[a]		Model 2[b]	
				Rate	95% CI	HR	95% CI	HR	95% CI
**MVPA×ST**									
HPA/LST	1,940	209	9,593	21.8	(19.0–24.9)	1.00	(Ref)	1.00	(Ref)
HPA/HST	1,445	190	7,114	26.7	(23.2–30.8)	1.06	(0.87–1.30)	1.03	(0.85–1.26)
LPA/LST	3,052	497	14,597	34.0	(31.2–37.2)	1.18	(1.00–1.38)	1.10	(0.94–1.30)
LPA/HST	3,088	738	13,900	53.1	(49.4–57.1)	1.41	(1.21–1.65)	1.25	(1.06–1.46)
Interaction[c]									
RERI						0.17	(-0.05–0.39)	0.11	(-0.11–0.33)
%RERI						41.7%		45.5%	
p-value						0.102		0.303	
**MVPA**									
High	3,385	399	16,707	23.9	(21.6–26.3)	1.00	(Ref)	1.00	(Ref)
Low	6,140	1,235	28,496	43.3	(41.0–45.8)	1.26	(1.12–1.42)	1.16	(1.03–1.30)
**ST**									
Low	4,992	706	24,190	29.2	(27.1–31.4)	1.00	(Ref)	1.00	(Ref)
High	4,533	928	21,014	44.2	(41.4–47.1)	1.18	(1.06–1.30)	1.11	(1.00–1.23)

CI, confidence interval; HR, hazard ratio; HST, high sitting time; HPA, high moderate-to-vigorous physical activity; LPA, low moderate-to-vigorous physical activity; LST, low sitting time; MVPA; moderate-to-vigorous physical activity; PY, person years; Ref, reference; RERI, Relative Excess Risk due to Interaction; ST, sitting times.

[a] Model 1: Adjusted for age, sex, and population density

[b] Model 2: Adjusted for model 1 and body mass index, smoking status, alcohol consumption, family structure, educational level, economic status, sleep duration, denture use, medication use, number of chronic disease count, and frailty status.

[c] The additive interaction was calculated as the RERI using the following equation: RERI = (HR [LPA/HST] − 1) − (HR [LPA/LST] + HR [HPA/HST] − 2). The values are shown as RERI (95% CI). It is significant ($p < 0.05$) if the 95% CI of the RERI is not above 0.

eTable 2. Results of sensitivity analysis for the relationship between moderate-to-vigorous physical activity and sitting time status and risk of long-term disability using a multiple imputation method for missing value of covariates

	n	Event	PY	Event/1,000 PY		Model 1[a]		Model 2[b]	
				Rate	95% CI	HR	95% CI	HR	95% CI
**MVPA×ST**									
HPA/LST	1,970	239	9,608	24.9	(21.9–28.2)	1.00	(Ref)	1.00	(Ref)
HPA/HST	1,478	223	7,128	31.3	(27.4–35.7)	1.08	(0.90–1.29)	1.03	(0.86–1.24)
LPA/LST	3,211	656	14,666	44.7	(41.4–48.3)	1.31	(1.13–1.52)	1.23	(1.06–1.43)
LPA/HST	3,505	1,155	14,059	82.2	(77.6–87.0)	1.73	(1.50–2.00)	1.52	(1.32–1.76)
Interaction[c]									
RERI						0.35	(0.15–0.54)	0.26	(0.06–0.45)
%RERI						47.2%		50.1%	
p-value						<0.001		0.004	
**MVPA**									
High	3,448	462	16,737	27.6	(25.2–30.2)	1.00	(Ref)	1.00	(Ref)
Low	6,716	1,811	28,724	63.0	(60.2–66.0)	1.48	(1.33–1.64)	1.37	(1.23–1.52)
**ST**									
Low	5,181	895	24,274	36.9	(34.5–39.4)	1.00	(Ref)	1.00	(Ref)
High	4,983	1,378	21,187	65.0	(61.7–68.6)	1.29	(1.18–1.40)	1.20	(1.10–1.31)

CI, confidence interval; HR, hazard ratio; HST, high sitting time; HPA, high moderate-to-vigorous physical activity; LPA, low moderate-to-vigorous physical activity; LST, low sitting time; MVPA; moderate-to-vigorous physical activity; PY, person years; Ref, reference; RERI, Relative Excess Risk due to Interaction; ST, sitting times.

[a] Model 1: Adjusted for age, sex, and population density.

[b] Model 2: Adjusted for model 1 and body mass index, smoking status, alcohol consumption, family structure, educational level, economic status, sleep duration, denture use, medication use, number of chronic disease count, and frailty status.

[c] The additive interaction was calculated as the RERI using the following equation: RERI = (HR [LPA/HST] − 1) − (HR [LPA/LST] + HR [HPA/HST] − 2). The values are shown as RERI (95% CI). It is significant ($p < 0.05$) if the 95% CI of the RERI is not above 0.

eTable 3. Results of sensitivity analysis for the relationship between moderate-to-vigorous physical activity and sitting time status and risk of long-term disability using the multivariable sub-distribution hazard model among older adults

	n	Event	PY	Event/1,000 PY		Model 1[a]		Model 2[b]	
				Rate	95% CI	SHR	95% CI	SHR	95% CI
**MVPA×ST**									
HPA/LST	1,970	239	9,608	24.9	(21.9–28.2)	1.00	(Ref)	1.00	(Ref)
HPA/HST	1,478	223	7,128	31.3	(27.4–35.7)	1.10	(0.92–1.31)	1.06	(0.89–1.27)
LPA/LST	3,211	656	14,666	44.7	(41.4–48.3)	1.32	(1.14–1.53)	1.23	(1.06–1.43)
LPA/HST	3,505	1,155	14,059	82.2	(77.6–87.0)	1.70	(1.48–1.97)	1.49	(1.29–1.73)
Interaction[c]									
RERI						0.28	(0.09–0.48)	0.21	(0.01–0.40)
%RERI						40.5%		41.9%	
p-value						<0.001		0.021	
**MVPA**									
High	3,448	462	16,737	27.6	(25.2–30.2)	1.00	(Ref)	1.00	(Ref)
Low	6,716	1,811	28,724	63.0	(60.2–66.0)	1.46	(1.31–1.61)	1.33	(1.20–1.48)
**ST**									
Low	5,181	895	24,274	36.9	(34.5–39.4)	1.00	(Ref)	1.00	(Ref)
High	4,983	1,378	21,187	65.0	(61.7–68.6)	1.27	(1.16–1.39)	1.19	(1.09–1.31)

CI, confidence interval; SHR, sub-distribution hazard ratio; HST, high sitting time; HPA, high moderate-to-vigorous physical activity; LPA, low moderate-to-vigorous physical activity; LST, low sitting time; MVPA; moderate-to-vigorous physical activity; PY, person years; Ref, reference; RERI, Relative Excess Risk due to Interaction; ST, sitting times.

[a] Model 1: Adjusted for age, sex, and population density

[b] Model 2: Adjusted for model 1 and body mass index, smoking status, alcohol consumption, family structure, educational level, economic status, sleep duration, denture use, medication use, number of chronic disease count, and frailty status.

[c] The additive interaction was calculated as the RERI using the following equation: RERI = (SHR [LPA/HST] − 1) − (SHR [LPA/LST] + SHR [HPA/HST] − 2). The values are shown as RERI (95% CI). It is significant ($p < 0.05$) if the 95% CI of the RERI is not above 0.
